# Scalable synthesis of (1-cyclopropyl)cyclopropylamine hydrochloride

**DOI:** 10.3762/bjoc.7.113

**Published:** 2011-07-21

**Authors:** Sergei I Kozhushkov, Alexander F Khlebnikov, Rafael R Kostikov, Dmitrii S Yufit, Armin de Meijere

**Affiliations:** 1 Institut für Organische und Biomolekulare Chemie der Georg-August-Universität Göttingen, Tammannstrasse 2, 37077 Göttingen, Germany, Fax: +49-551-399475; 2 Department of Chemistry, Saint Petersburg State University, Universitetskii Prosp. 26, Petrodvorets, 198504 St. Petersburg, Russia; 3 Department of Chemistry, University of Durham, South Rd., Durham DH1 3L, UK

**Keywords:** amines, building blocks, carboxylic acids, Curtius degradation, cyclopropanes

## Abstract

1-Cyclopropylcyclopropanecarboxylic acid (**2**), which is accessible on a large scale (900 mmol) from 1-bromo-1-cyclopropylcyclopropane (**1**) in 64% yield (89% on a 12.4 mmol scale), has been subjected to a Curtius degradation employing the Weinstock protocol to furnish the *N*-Boc-protected (1-cyclopropyl)cyclopropylamine **3** (76%). Deprotection of **3** with hydrogen chloride in diethyl ether gave the (1-cyclopropyl)cyclopropylamine hydrochloride (**4**·HCl) in 87% yield.

## Introduction

Several recent patent applications have stirred an increasing interest in research departments of pharmaceutical and agrochemical companies concerning 1- and 2-substituted 1,1'-bicyclopropyl derivatives. Among them, intermediates containing a (1-cyclopropyl)cyclopropylamine moiety appear to be particularly important and desirable for the preparation of biologically active and pharmacologically relevant compounds. For example, a number of derivatives of (1-cyclopropyl)cyclopropylamine (**4**) have been found to be useful variously for the treatment of hepatitis C [3–4], as pest control agents [5], as inhibitors of methicillin-resistant *Staphylococcus aureus* [5], as pesticides, insecticides and acaricides [7–13] and more. This amine has been prepared from cyclopropyl cyanide [3–13] by application of the Szymoniak–Kulinkovich reductive cyclopropanation procedure [14–15]. In our hands, however, this patented protocol [3–13] provided poor yields (15–20%) of impure **4** [16], which had to be purified by conversion to the corresponding *tert*-butyl carbamate and subsequent column chromatography. Thus, this procedure was not easily scalable to 10–50 g quantities. To meet such demands, we have developed an alternative route to **4** from the easily available corresponding carboxylic acid **2** [17–18] by Curtius degradation [19–20].

## Results and Discussion

**Scheme 1:**
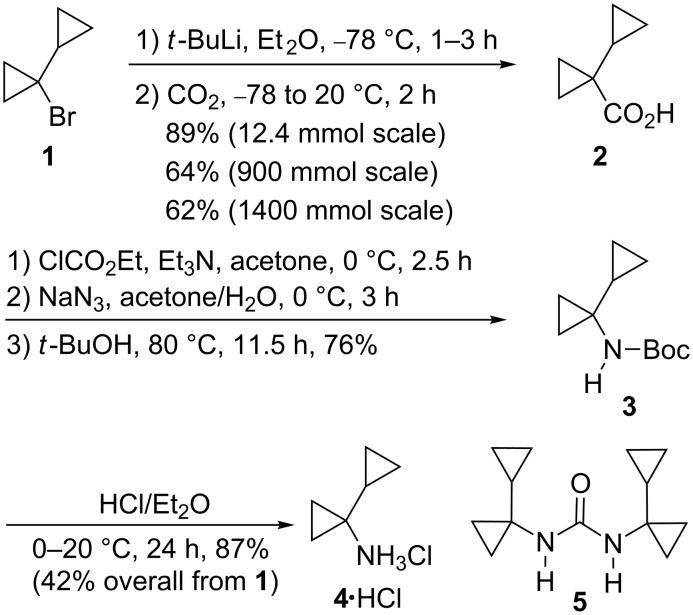
Preparation of 1-(cyclopropyl)cyclopropylamine hydrochloride (**4**·HCl).

Preparation of the acid **2** from the known 1-bromo-1-cyclopropylcyclopropane (**1**) [21–22] according to the published procedure [17] was accomplished on a 100 g scale (Scheme 1). However, the yield of the carboxylation on a scale of 12.4 mmol, 900 mmol and 1400 mmol, was 89, 64 and 62%, respectively. This is associated with the longer reaction time employed on a larger scale, during which the intermediate 1-cyclopropyl-1-lithiocyclopropane may be trapped by the by-product *tert*-butyl bromide, leading to isobutene by dehydrobromination [23–24]. Indeed, the reaction on a 200 mmol scale, but over a period of 3 h, furnished **2** in 46% yield only. According to previous experience, this undesired side reaction can be suppressed by employing two equivalents of *tert*-butyllithium [23]. Thus, the yield of **2** may be improved even for large scale preparation.

**Figure 1:**
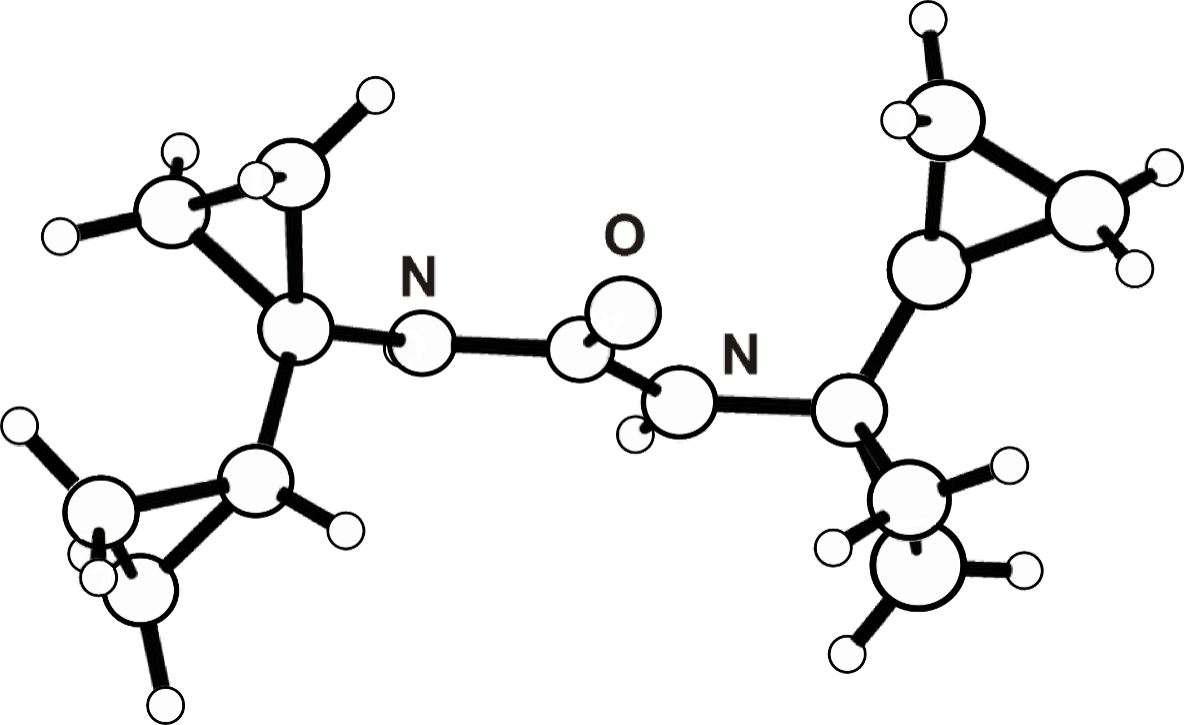
Structure of 1,3-di(bicyclopropyl)urea (**5**) in the crystal [26].

Curtius degradation of the acid **2** via the corresponding azide, according to the Weinstock protocol [19–20] as previously employed in different examples [2,25], furnished the *N*-Boc-protected (1-cyclopropyl)cyclopropylamine **3** in 76% yield. It was essential to carefully dry the solution of the intermediate azide, otherwise the yield of **3** dropped dramatically, and the desired product was accompanied by 1,3-di(bicyclopropyl)urea (**5**) in up to 50% yield (Scheme 1). The structure of the latter was confirmed by an X-ray crystal structure analysis (Figure 1) [26].

The carbamate **3** was deprotected by treatment with hydrogen chloride in diethyl ether affording the amine hydrochloride **4**·HCl in 87% yield. The latter was thus obtained from 1-bromo-1-cyclopropylcyclopropane (**1**) on a scale of 50 g in 42% overall yield (Scheme 1).

## Conclusion

The newly developed procedure allows the preparation of 1-(cyclopropyl)cyclopropylamine (**4**) in five steps from commercially available methyl cyclopropanecarboxylate, reproducibly, on a 50 g and even larger scale. In this respect it is superior to the previously published and patented access to **4** from cyclopropanecarbonitrile, which in the hands of five different researchers in our laboratory required chromatographic separation of the intermediately prepared *N*-Boc derivative, which involved the rather costly di-*tert*-butyl pyrocarbonate and made that an overall three-step procedure.

## Experimental

^1^H and ^13^C NMR spectra were recorded at 300 MHz [^1^H] and 62.9 MHz [^13^C, additional DEPT (Distortionless Enhancement by Polarization Transfer)] on Bruker AM 250 and Varian Mercury Vx300 instruments in CDCl_3_ and D_2_O solutions, CHCl_3_/CDCl_3_ and DHO as internal references. EI-MS, ESI-MS and HRMS spectra were measured with Finnigan MAT 95 (70 eV), Finnigan LCQ and Bruker Daltonic APEX IV 7T FTICR instruments, respectively. Melting points were determined on a Büchi 510 capillary melting point apparatus, values are uncorrected. TLC analyses were performed on precoated sheets (0.25 mm Sil G/UV254) from Macherey-Nagel). All chemicals were used as received. 1-Bromo-1-cyclopropylcyclopropane (**1**) was obtained according to the previously published procedure [21]. A 5.0 N solution of HCl in Et_2_O was prepared by saturation of anhydrous Et_2_O with gaseous HCl at 0 °C. Anhydrous diethyl ether was obtained by distillation from sodium benzophenone ketyl, acetone by distillation from anhydrous potassium carbonate. Anhydrous *tert*-butyl alcohol was obtained employing molecular sieves (4 Å) [27]. Organic extracts were dried over MgSO_4_. All reactions in anhydrous solvents were carried out under an argon atmosphere in flame-dried glassware.

### Synthesis of 1-cyclopropylcyclopropanecarboxylic acid (**2**)

Under mechanical stirring and cooling with pentane/liq. N_2_, a solution of *t*-BuLi (1.7 M in pentane, 560 mL, 952.0 mmol) was added dropwise to a solution of 1-bromo-1-cyclopropylcyclopropane (**1**) (146.0 g, 907.0 mmol) in anhydrous Et_2_O (2.2 L) at −78 °C within 40 min. After stirring at −78 °C for an additional 25 min, an excess of dry ice was added in several portions (T ≤ −70 °C), and the mixture was allowed to slowly warm up to ambient temperature during a period of 2 h. The reaction was quenched with an ice-cold solution of KOH (60.0 g, 1.070 mol) in H_2_O (1 L), the aqueous layer was washed with ether (3 × 100 mL), and then acidified with conc. aq. HCl solution at 0–5 °C (ca. 175 mL). The resulting mixture was extracted with ether (4 × 300 mL), the combined organic phases were dried and concentrated under reduced pressure to give the acid **2** (73.2 g, 64%) as colorless crystals, mp 50–51 °C (lit. [17]: mp: 51–52 °C), which was used in the next step without further purification. Its NMR spectra were identical to the published ones [17].

### Synthesis of *tert*-butyl 1-(cyclopropyl)cyclopropylcarbamate (**3**)

To a mechanically stirred solution of the acid **2** (70.60 g, ca. 560.0 mmol) in anhydrous acetone (1.7 L), was added Et_3_N (76.2 g, 105.0 mL, 753.0 mmol) dropwise at −5 °C. After additional stirring at this temperature for 15 min, neat ethyl chloroformate (103.7 g, 91.0 mL, 956.0 mmol) was added at the same temperature over a period of 30 min, and the resulting mixture was stirred at this temperature for an additional 2 h. Then a solution of NaN_3_ (75.0 g, 1.0 mol) in H_2_O (200 mL) was added over a period of 1.5 h. The reaction mixture was stirred at 0 °C for 1.5 h, concentrated under reduced pressure at 0 °C to about a half of the original volume, poured into ice-cold water (2 L), and the mixture extracted with diethyl ether (4 × 400 ml) and pentane (2 × 350 ml). The combined organic solutions were washed with ice-cold water (2 × 400 mL), dried under stirring with MgSO_4_ at 0 °C for 1 h and concentrated under reduced pressure at 0 °C/20–30 Torr. The residue was taken up with pentane (300 mL), dried and concentrated under the same conditions. It was then dissolved in anhydrous *t*-BuOH (200 mL), and this solution was added dropwise to anhydrous *t*-BuOH (1300 mL) kept at 80 °C under vigorous stirring over a period of 2.5 h. The resulting solution was heated under reflux for an additional 9 h. The main volume of *t*-BuOH (ca. 1300 mL) was distilled off under ambient pressure in a nitrogen flow. After cooling, the residue mixture was dried at 20 °C/0.1 Torr to give essentially pure carbamate **3** (84.0 g, 76%) as a colorless solid, mp 69–70 °C, *R*_f_ 0.38 (hexane/Et_2_O 5:1), which was used in the next step without further purification. ^1^H NMR (300 MHz, CDCl_3_) δ 4.91 (br s, 1H, NH), 1.39 (s, 9H, 3 CH_3_), 1.30–1.20 (br m, 1H, *c*Pr-H), 0.64–0.57 (br m, 2H, *c*Pr-H), 0.52–0.45 (br m, 2H, *c*Pr-H), 0.37–0.31 (m, 2H, *c*Pr-H), 0.09–0.04 (m, 2H, *c*Pr-H); ^13^C NMR (62.9 MHz, CDCl_3_) δ 155.2 (C), 79.0 (C), 34.1 (C), 28.3 (3 CH_3_), 15.6 (CH), 11.9 (2 CH_2_), 2.6 (2 CH_2_); EIMS (70 eV) *m*/*z*: 141 (M^+^ − C_4_H_8_), 126, 96, 82, 58, 57, 43; HRMS–ESI (*m*/*z*): calcd for C_11_H_19_NNaO_2_, 220.1308; found, 220.1314.

### Synthesis of (1-cyclopropyl)cyclopropylamine hydrochloride (**4**·HCl)

Under stirring, a solution of the carbamate **3** (84.0 g, 425.8 mmol) in Et_2_O (100 mL) was added to a ca. 5.0 N HCl solution in Et_2_O (700 mL) in one portion at 0 °C. The reaction mixture was stirred at 0 °C for 4 h and at ambient temperature for 20 h. The formed precipitate was filtered off, washed with Et_2_O (200 mL) and dried in a vacuum desiccator over P_4_O_10_ overnight to give **4**·HCl (49.7 g, 87%) as a colorless powder, which slowly decomposes above ca. 135 °C and melts at 196–198 °C (dec.); ^1^H NMR (300 MHz, D_2_O) δ 1.30–1.26 (m, 1H, *c*Pr-H), 0.71–0.60 and 0.60–0.55 (m AA'BB', 4H, *c*Pr-H), 0.49–0.42 and 0.13–0.08 (m AA'BB', 4H, *c*Pr-H).

When a solution of the intermediate azide in the preparation of **3** was not sufficiently dried, the thermolysis in *t*-BuOH along with *tert*-butylcarbamate **3** gave the 1,3-di(bicyclopropyl)urea (**5**) in up to 50% yield. Compound **5** was isolated as a colorless solid after deprotection of **3** with HCl/Et_2_O by evaporation of the mother liquor followed by recrystallization of the residue from hexane/CHCl_3_; mp 159–161 °C. The structure of **5** was confirmed by X-ray crystal structure analysis [26]. **5**: ^1^H NMR (300 MHz, CDCl_3_) δ 5.21 (br s, 2H, NH), 1.28–1.16 (m, 2H, 2 CH *c*Pr-H), 0.73–0.61 (m AA'BB', 8H, 4 CH_2_, *c*Pr-H), 0.44–0.41 and 0.17–0.13 (m AA'BB', 8H, 4 CH_2_, *c*Pr-H); ^13^C NMR (62.9 MHz, CDCl_3_) δ 158.8 (C), 33.9 (2 C), 15.5 (2 CH), 12.6 (2 CH_2_), 2.8 (6 CH_2_); EIMS (70 eV) *m*/*z*: 219 (M^+^ − H), 205 (M^+^ − H−CH_2_), 191 (M^+^ − H−C_2_H_4_), 124 (M^+^ − H−NC_6_H_9_), 96 (M^+^ − H−NC_6_H_9_−CO), 82 (M^+^ − H−NC_6_H_9_−CH_2_−CO).
